# Partner phubbing and relational aggression in romantic relationships among young adults in China: the roles of social support and gender

**DOI:** 10.3389/fpsyg.2024.1470175

**Published:** 2025-01-10

**Authors:** Liang Ying, Lvzhou Ren, Xin Wang, Jiankang He, Xue Yang, Guohua Zhang

**Affiliations:** ^1^School of Renji, Wenzhou Medical University, Wenzhou, China; ^2^College of Teacher Education, Zhejiang Normal University, Jinhua, China; ^3^School of Psychology, Nanjing Normal University, Nanjing, China; ^4^Center for Health Behaviours Research, JC School of Public Health and Primary Care, Faculty of Medicine, The Chinese University of Hong Kong, Shatin, Hong Kong SAR, China; ^5^Department of Psychology, School of Mental Health, Wenzhou Medical University, Wenzhou, China; ^6^Zhejiang Provincial Clinical Research Center for Mental Health, The Affiliated Wenzhou Kangning Hospital, Wenzhou Medical University, Wenzhou, China; ^7^Wenzhou Key Laboratory of Basic and Translational Research for Mental Disorders, Wenzhou, China

**Keywords:** romantic relationships, partner phubbing, relational aggression, social support, gender, young adults

## Abstract

**Introduction:**

Phubbing may have significant interpersonal consequences. This study examines the association between partner phubbing and relational aggression, the mediation effect of social support, and the moderation effect of gender among young Chinese adults.

**Method:**

A total of 772 young adults currently in a romantic relationship participated in an online survey that assessed their partner phubbing, relational aggression, and social support (i.e., ideal support, actual support, and discrepancy between ideal and actual support).

**Results:**

The results show that partner phubbing was positively and significantly correlated with relational aggression for males and females, respectively. Actual support and support discrepancy partially mediated the relationship between partner phubbing and relational aggression among all participants. Regarding gender difference, actual support and support discrepancy partially mediated the relationship between partner phubbing and relational aggression in females but were not significant mediators for males.

**Discussion:**

These findings suggest that partner phubbing had a significant effect on relational aggression in romantic relationships for both male and female participants. Social support may play a significant role between partner phubbing and relational aggression in females only. Tailored interventions for partner phubbing to prevent negative interpersonal consequences are warranted.

## 1 Introduction

Romantic relationships have become an indispensable part of late adolescence and early adulthood in terms of crucial tasks of self-identity, academic achievement, sexuality, and future planning (Furman and Buhrmester, 1992). In recent decades, conflicts and aggression within young adults’ romantic relations have been a central focus of studies in this field (e.g., Deans and Bhogal, 2019; Luetke et al., 2020; Voulgaridou and Kokkinos, 2023). However, little is known about the manifestations and factors of relational aggression in Chinese young adults’ romantic relationships. Relational aggression is defined as a type of aggression that is intended to harm others through deliberate manipulation of their social standing and relationships (Steinberg et al., 2001). It is an indirect damage that contributes to unsatisfactory friendships, feelings of rejection, or group exclusion (Crick and Grotpeter, 2010). It includes relational aggression perpetration and victimization (Ellis et al., 2009; Li, 2013) and is a different construct from psychological and verbal aggression and often happens without physical aggression. Relational aggression is a much broader category that includes behaviors such as verbal insults (e.g., rumor spreading, embarrassment), intimidation, threats, coercion, and accusations (Kasian and Painter, 1992; Murphy et al., 1993), which has been linked to mental problems (e.g., loneliness, depression, and substance abuse) (Bagner et al., 2007) and poor quality of interpersonal relationships (e.g., having high level of antisocial, borderline personality, trusting problems and jealousy) (Linder et al., 2002; Goldstein et al., 2008; Leadbeater et al., 2008).

Studies have shown that social-cognitive factors, relationship characteristics, traits and dispositions, and psychological status are four important domains of risk factors of relational aggression (e.g., Crick & Grotpeter, 2010; Bailey and Ostrov, 2008). Cyber technology provides a new venue for the expression of relational aggression for youngsters. At present, the increasing use of cyberspace creates a new medium for youth to become perpetrators and victims of peer aggression (Dempsey et al., 2009). In actuality, one study found that “cyber,” “overt” and “relational” represent distinct subtypes of aggressive behavior in cyberspace (Dempsey et al., 2011). Partner phubbing may be an important factor affecting relational aggression among young adults in the digital age. However, the mechanism behind the relationship remains unclear.

### 1.1 Association between partner phubbing and relational aggression in romantic relationships

Partner phubbing refers to snubbing and ignoring one’s significant other in the context of social contact by focusing one’s attention on a mobile phone (Ugur and Koc, 2015; Abeele et al., 2016). It was reported that 25% of married couples and 42% of unmarried partners are occupied by a smartphone when they have physical connections (Lenhart et al., 2015). Individuals with phubbing behavior are more likely to ignore their partner’s needs, their partner may then experience higher sense of rejection and exclusion (Abeele et al., 2016; McDaniel and Coyne, 2016; Roberts and David, 2016; Chotpitayasunondh and Douglas, 2018a), and lower levels of trust and social emotional closeness (Przybylski et al., 2013; Abeele et al., 2016; Misra et al., 2016; Roberts and David, 2016). On the other hand, individuals who perceive that they have to compete with a machine device to get attention from the person who is supposed to love them are more likely to have reduced feelings of belongingness that go on to affect their perceived quality of communication relationship (Chotpitayasunondh and Douglas, 2018b).

The cognitive appraisal theory indicates that individuals’ behaviors do not stem directly from a stress event, but rather from the subjective cognitive appraisal of that event (Lazarus, 1991). Partner phubbing can be cognitively appraised as a stressful interpersonal event that threatens harm or loss to the individual. This threatening appraisal elicits romantic jealousy as an emotional response. In turn, romantic jealousy supplies emotional motivation for relational aggression, serving as a behavioral attempt to cope with the stressful situation of partner phubbing (Buller et al., 2022; Wang et al., 2022; Wang et al., 2024). Evidence has accumulated that partner phubbing predicts lower relationship quality (Carnelley et al., 2023) and relationship satisfaction (Wang et al., 2021). Romantic relationship satisfaction is a predictor of partner violence (Zhan et al., 2022). Recently, a study showed that partner phubbing was significantly and positively related to young adults’ relational aggression in romantic relationship (Wang et al., 2024). Another study found that peer phubbing had a significant positive predictive effect on cyber dating abuse perpetration and victimization (Dai et al., 2024). Based on the literature review and theoretical derivation, this study hypothesizes that partner phubbing is positively associated with relational aggression in romantic relationships (H1).

### 1.2 Social support from partner as mediators

Social support refers to resources provided by others, which is also regarded as the function and quality of social relationships (Thoits, 2011). Newcomb (1990) distinguished two dimensions of social support: (1) actual support, which is actual help received within a given time frame; and (2) ideal support, which pertains to the anticipation of help in time of need. Some researchers have noted a third type of social support, namely support discrepancy, which is the gap between actual and ideal support (Power et al., 1988). Based on the main effect model of social support (Finney et al., 1984), social support, as a protective factor, can significantly diminish adverse outcomes for individuals (e.g., stress and depression). It is possible that partners with high actual support and/or low support discrepancy in romantic relationships are less likely to have interpersonal conflicts or problems (in the context of this study, relational aggression). This study summarizes the previous research and deduces the possible mediation effects of actual support and support discrepancy on partner phubbing and relational aggression in romantic relationships.

Firstly, romantic relationships are shared by both parties, but phubbing behavior may lead to more avoidance of face-to-face chatting and less engage in self-disclosure and meaning seeking in reality. That is, individuals who frequently exhibits phubbing behaviors are more likely to use phubbing behavior to monitor information from the outside world and escape from the real social environment and seek more social connections on social networks to create a sense of being in a group (Zhan et al., 2022). In a romantic relationship, partner phubbing would decrease the interaction between partners and further reduce actual support from the partner. A previous study reported the mediation role of social support in the relationship between internet addiction and aggression (Gao et al., 2016). Therefore, it is reasonable to assume that actual social support would mediate the relationship between partner phubbing and relational aggression in romantic relationships (H2).

Secondly, according to the social support class compensation model, relatives (in this case, romantic partners) are regarded as the core resources of support, and the model implies that people tend to have high expectations for support from their partners (Cantor and Little, 1985). However, prolonged use of smartphones or other electronic products in social settings and disconnecting from each other is likely to prevent partners from exchanging social support, which results in deviation from expectations. Hence, the present study hypothesizes that the association between partner phubbing and relational aggression is mediated by the discrepancy between actual and ideal support among partners (H3).

### 1.3 Gender differences in the proposed mediation model

Gender is nevertheless an important factor that influences the consequences of phubbing behaviors. Through the lens of cognitive appraisal theory (Lazarus, 1991), how both parties subjectively construe and evaluate phubbing impacts resulting behavioral response, such as reduce actual support and increase the discrepancy between actual and ideal support, and destructive conflict responses like relational aggression. The same partner phubbing behaviors may be interpreted differently by males and females, eliciting divergent emotional and behavioral responses (Wang et al., 2024).

Few empirical examinations of the differential effects of partner phubbing by gender exist, and the results are inconsistent. For instance, some studies have reported that females scored higher than males on measures of phubbing (e.g., Chotpitayasunondh and Douglas, 2016; Balta et al., 2018; Błachnio and Przepiorka, 2018), while others have found that phubbing scores for male college students were significantly higher than for their female counterparts (e.g., Chi et al., 2022). A recent study has indicated that partner phubbing and romantic jealousy was significant in female group, but not in male group; partner phubbing and romantic jealousy was significant in female group, but not in male group (Wang et al., 2024). Similarly, another study has concluded that women’s higher levels of attachment anxiety is significantly associated with higher ratings of perceived partner phubbing, while men’s higher levels of attachment avoidance is significantly associated with higher ratings of perceived partner phubbing (Bröning and Wartberg, 2022).

Overall, it is important to pay attention to gender when examining the impact of partner phubbing on social support and relational aggression. According to the literature review and theoretical foundations, we will not make specific assumptions here. We hypothesize that there were differences in the relationship among partner phubbing, social support, and relational aggression (H4).

## 2 Methods

### 2.1 Participants and procedures

Data were collected from unmarried young adults in China. The inclusion criteria included: (1) aged 18 ~ 35 years old, which was seen as young adults in China; (2) speaker of Chinese; (3) uses a smartphone daily; (4) currently involved in a romantic relationship. The survey was conducted in February 2021 through Wen Juan Xing^1^, a web-based survey platform widely used in China. The respondents were recruited through convenience sampling by means of WeChat groups and circles of friends. WeChat is a social networking service frequently used by Chinese people in almost all age groups, but especially by young adults. Sending Wen Juan Xing links or a “quick response code” (QR code) via WeChat has become an important way to get access to eligible subjects and collect research data.

Participants read an informed consent document before beginning the online survey and were given RMB 5 (about USD 0.77) cash through Alipay to compensate for their time spent. Of the 833 responses from young adults who completed the survey anonymously, 7.44% (62) were excluded from the data analysis because of incorrect answers to items designed to detect whether respondents were answering questions seriously (such as “Please select ‘Never’ for this question”). The remaining 92.68% of responses (772) were used in the analysis reported below. Ethical approval was obtained from the local school ethics committee.

### 2.2 Measures

#### 2.2.1 Partner phubbing

Phubbing in a romantic relationship was assessed by the 9-item Partner Phubbing Scale developed by Roberts and David (2016). Items were rated on a 5-point Likert scale ranging from 1 (never) to 5 (all the time), with higher scores reflecting higher levels of partner phubbing. The scale was used in the Chinese population and showed good reliability and validity (Ding et al., 2020). In this study, the Cronbach’s *α* of the scale was 0.76.

#### 2.2.2 Social support

Perceived social support was measured using the 10-item Significant Others Scale in romantic relationships (Power et al., 1988; Zhang et al., 2011). Items were rated using a 7-point Likert scale ranging from 1 (never) to 7 (always), with higher scores indicating higher levels of social support from the partner in a romantic relationship. Participants were asked to complete two versions of the scale with same items about support in different aspects. One version, labeled ‘actual’ support, asked them to respond according to their actual condition; the other version, labeled ‘ideal’ support, asked them to respond according to their expectation of support. The support discrepancy variable was created by deducting the score for actual support from the score for ideal support. The Chinese version of the scale has shown good reliability and validity (Zhang et al., 2011). In this study, the Cronbach’s *α* values of the actual support scale and the ideal support scale were 0.92 and 0.93, respectively.

#### 2.2.3 Relational aggression

The Chinese version of 10-item Dating Relational Aggression Subscale was used to measure experiences of relational aggression in romantic relationships in the last 3 months (Ellis et al., 2009; Li, 2013). The scale contains two subscales: relational aggression perpetration subscale and victimization subscale. Responses were made on a 5-point scale ranging from 1 (not at all true) to 5 (very true). Example items include “My girlfriend or boyfriend ignores me when they are angry” and “When I’m angry at my girlfriend or boyfriend, I try to make him jealous.” The total score ranged from 10 to 50, with higher scores indicating higher levels of relational aggression. The scale is suitable for evaluating the level of relational aggression among young people in China (Li, 2013; Zhao et al., 2024). In this study, the Cronbach’s α of relationship aggression victimization and perpetration were 0.74 and 0.75, respectively.

### 2.3 Data analysis

Descriptive and correlation analyses among all variables were carried out by gender. We constructed a parallel multiple mediation model using an independent variable (partner phubbing), three mediators (i.e., ideal support, actual support, and discrepancy between ideal and actual support), and a dependent variable (relational aggression). The mediation analysis was conducted using the PROCESS macro (Model 4; Hayes, 2017). Bootstrapping analysis based on 5,000 resamples was used whereby a 95% confidence interval (CI) without 0 reflected statistical significance. The proportion mediated (PM) was used to evaluate the effect sizes of the mediators. Background variables, namely gender, age, time spent on mobile phone use, and duration of the romantic relationship, were controlled in the regression models, as these are variables that have been found to be significantly correlated to social support and relational aggression (e.g., Coyne et al., 2017). The level of statistical significance was set at *p* < 0.05, and the analyses were performed using SPSS 26.0.

## 3 Results

### 3.1 Descriptive characteristics and preliminary analysis

Data from the participants who completed the survey are reported in this study (*N* = 772; 34.97% male). The background characteristics of the subjects (gender, family origin, one-child family, time spent on mobile phone per day, long-distance relationship, education, age, relationship duration, and situations of phubbing) are described in Table 1. Males reported a higher level of the support discrepancy than female (*t* = 2.38, *p* < 0.05); however, gender differences in partner phubbing, relational aggression, ideal support, and actual support were not significant (*p* > 0.05).

**Table 1:** Background characteristics of the participants (*N* = 772).

Variable	*N*	%	Variable	*N*	%
Gender			Time spent on mobile phone per day (hours)		
Male	270	34.97	< 2	8	1.04
Female	502	65.03	2 ~ 4	76	9.84
Age			4 ~ 6	234	30.31
≤18	54	6.99	6 ~ 8	256	33.16
19	82	10.63	>8	198	25.65
20	205	26.55	Long-distance relationship		
21	135	17.49	Yes	356	46.11
22	98	12.69	No	416	53.89
23	59	7.64	Relationship duration		
24	49	6.35	<1 month	58	7.51
25 ~ 35	90	11.66	1 ~ 3 months	122	15.80
Family origin			3 ~ 6 months	101	13.08
Urban	530	68.65	6 months ~1 year	108	13.99
Rural	242	31.35	1 ~ 3 years	248	32.12
One-child family			Situations of phubbing		
One child	313	40.54	Having a meal	244	31.61
More than one child	459	59.46	Standing in a queue	330	42.75
Education			Waiting for a film to start	195	25.26
Middle school and below	2	0.26	Waiting for a vehicle	303	39.25
High school	23	2.98	Taking a vehicle	309	40.03
Bachelor’s and junior college	648	83.94	Taking a walk	62	8.03
Master’s or doctoral	99	12.82			

Table 2 shows the mean (*M*), standard deviation (*SD*), and correlations for all the study variables across gender. For the male participants, PP was positively and significantly correlated with SD (*r* = 0.14, *p* < 0.05) and RA (*r* = 0.28, *p* < 0.001). RA was negatively and significantly correlated with AS (*r* = −0.25, *p* < 0.001) and IS (*r* = −0.22, *p* < 0.001). For female participants, PP was positively and significantly correlated with SD (*r* = 0.16, *p* < 0.001) and RA (*r* = 0.30, *p* < 0.001), and negatively and significantly correlated with AS (*r* = −0.17, *p* < 0.001). RA was negatively and significantly correlated with AS (*r* = −0.31, *p* < 0.01) and IS (*r* = −0.27, *p* < 0.001). The correlation between SD and RA was marginally significant (*r* = 0.09, *p* = 0.058).

**Table 2:** Descriptive statistics and correlations among variables (*N* = 772).

Male	*M*	*SD*	1	2	3	4	5	*M*	*SD*	Female
1. PP	26.90	6.05		−0.17^***^	−0.06	0.16^***^	0.30^***^	27.56	5.68	1. PP
2. AS	53.03	10.81	−0.05		0.73^***^	−0.45^***^	−0.31^***^	52.20	10.33	2. AS
3. IS	56.56	10.47	0.05	0.76^***^		0.28^***^	−0.27^***^	57.05	9.60	3. IS
4. SD	3.53	7.37	0.14^*^	−0.39^***^	0.31^***^		0.09^+^	4.85	7.34	4. SD
5. RA	22.12	7.26	0.28^***^	−0.25^***^	−0.22^***^	0.07		21.91	7.13	5. RA

PP, partner phubbing; AS, actual support; IS, ideal support; SD, support discrepancy; RA, relational aggression. The descriptive statistics and correlations among variables for the male participants are in the bottom left corner, and those for the female participants are in the top right corner. ^+^*p* < 0.10, *p < 0.05, ***p < 0.001.

### 3.2 Mediation model

**Figure 1:**
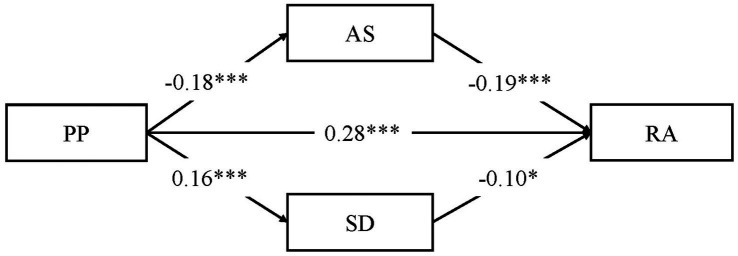
Mediation effect of social support among all participants (*N* = 772).

The correlation between PP and IS was not significant and IS was therefore not included as a mediator in the mediation model. Among the participants, the total effect of PP on RA was positive and significant (*β* = 0.31, *t* = 8.97, *p* < 0.001). As Figure 1 shows, after the addition of AS and SD as mediation variables, PP was significantly and positively associated with RA (*β* = 0.28, *t* = 8.35, *p* < 0.001) and SD (*β* = 0.16, *t* = 4.34, *p* < 0.001), but significantly and negatively associated with AS (*β* = 0.14, *t* = 4.05, *p* < 0.001). AS (*β* = 0.28, *t* = 7.32, *p* < 0.001) and SD (*β* = 0.09, *t* = 2.33, *p* < 0.05) were significantly and negatively associated with RA.

As Table 3 shows, AS partially mediated the association between PP and RA (B = 0.04, SE = 0.01, 95% CI [0.01, 0.07]), the mediation effect accounting for 12.7% of the total effect. SD partially mediated the relationship between PP and RA (B = −0.01, SE = 0.01, 95% CI [−0.03, −0.01]), the mediation effect accounting for 4.3% of the total effect. The model explained 17.0% of the variance of RA in total.

**Table 3:** Mediation effect of social support on the association between partner phubbing and relational aggression among all participants (*N* = 772).

Path	Estimate	*SE*	95% CI	Account PM (%)
PP → AS→RA	0.04	0.01	[0.01, 0.07]	12.7
PP → SD → RA	−0.01	0.01	[−0.03, −0.01]	4.3
PP → RA	0.28	0.03	[0.22, 0.35]	17.0

PM, proportion mediated.

### 3.3 Gender difference in the mediation model

**Figure 2:**
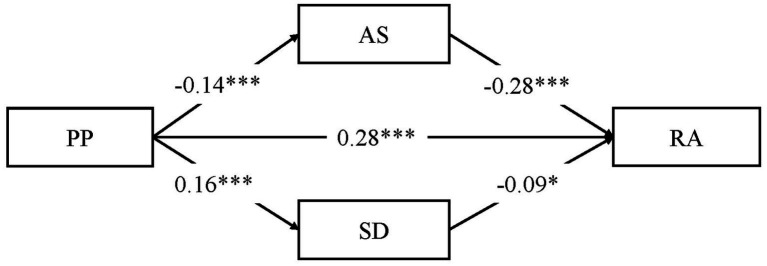
Mediation effect of social support among female participants (*N* = 502).

For female participants, PP was significantly and positively associated with RA (*β* = 0.32, *t* = 7.44, *p* < 0.001). As Figure 2 shows, after the addition of AS and SD as mediation variables, PP was significantly and positively associated with RA (*β* = 0.28, *t* = 6.68, *p* < 0.001) and SD (*β* = 0.16, *t* = 3.64, *p* < 0.001), but significantly and negatively associated with AS (*β* = −0.18, *t* = 4.13, *p* < 0.001). AS (*β* = −0.19, *t* = 6.11, *p* < 0.001) and SD (*β* = −0.10, *t* = 2.08, *p* < 0.05) were significantly and negatively associated with RA. As Table 4 shows, AS partially mediated the relationship between PP and RA (B = 0.05, SE = 0.02, 95% CI [0.02, 0.08]), and the mediation effect accounted for 16.0% of the total effect. SD partially mediated the relationship between PP and RA (B = −0.02, SE = 0.01, 95% CI [−0.03, −0.01]), and the mediation effect accounted for 4.9% of the total effect. The model explained 21.0% of the variance of relational aggression in total.

**Table 4:** Mediation effect of social support between partner phubbing and relational aggression among female participants (*N* = 502).

Path	Estimate	SE	95% CI	Account PM (%)
PP → AS→RA	0.05	0.02	[0.02, 0.08]	16.0
PP → SD → RA	−0.02	0.01	[−0.03, −0.01]	4.8
PP → RA	0.28	0.04	[0.20, 0.36]	21.0

**Figure 3:**
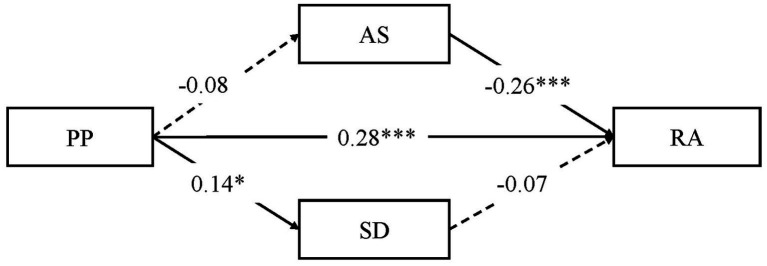
Mediation effect of social support among male participants (*N* = 270).

Figure 3 shows the mediation effect model for the male participants. PP was positively associated with RA (*β* = 0.28, *t* = 4.87, *p* < 0.001) and SD (*β* = 0.14, *t* = 2.33, *p* < 0.05). AS was negatively associated with RA (*β* = −0.26, *t* = 4.06, *p* < 0.001). Other associations were not significant. No significant mediator was found for the male participants.

## 4 Discussion

The current study proposed a mediating model to test the underlying mechanisms in the association between partner phubbing and relational aggression across gender among Chinese young adults. We found that partner phubbing was significantly associated with relational aggression perpetration and victimization in romantic relationships for both male and female participants, but that social support mediated this relationship in female participants only.

### 4.1 Partner phubbing and relational aggression

We found a positive association between partner phubbing and relational aggression; H1 is therefore supported. This result is consistent with prior studies (e.g., McDaniel and Coyne, 2016; Wang et al., 2017; Wang et al., 2024). Phubbing is considered a risk factor for problematic phone use, and it has been shown to affect psychological well-being (Lai et al., 2022). Partner phubbing directly affects the quality of interaction, communication, and relationship satisfaction (Miller-Ott and Kelly, 2015; McDaniel and Coyne, 2016; Roberts and David, 2016; Rotondi et al., 2017), which may increase conflict and relational aggression between partners in romantic relationships.

These results corroborate the cognitive appraisal theory (Lazarus, 1991), which suggests that phubbing behaviors may disturb communication between partners, thereby threatening the quality of interaction and relationship satisfaction (Abeele et al., 2016; Halpern and Katz, 2017; McDaniel and Coyne, 2016; Wang et al., 2017), leading to relational aggression. Empirical research has found that, use or were distracted by smartphones when dating or interacting may be perceived as exclusion and neglect by their partners, this may lead to young adults’ dating violence perpetration and victimization (Linder et al., 2002; Schacter et al., 2019). The present study thus confirms that partner phubbing has a significant effect on relational aggression perpetration and victimization in romantic relationships. This result provides a new perspective on intervention and prevention of relational aggression in romantic relationships. Given that partner phubbing may increase relational aggression, young adults need to enhance intimacy as the fundamental element of their romantic relationship and strengthen self-disclosure of information to one another to build trust (e.g., Chotpitayasunondh and Douglas, 2016), especially when they have to use mobile phones during dating.

### 4.2 Actual support and support discrepancy as mediators

We found that actual support partially mediated the positive relationship between partner phubbing and relational aggression, whereas support discrepancy suppressed such an association; H2 and H3 are partially supported. On the one hand, partner phubbing enhances relational aggression by reducing actual support; that is, phubbing behavior may reduce actual support to the partner, causing their dissatisfaction with the relationship and leading to relational aggression. Therefore, enhancing the level of actual support would decrease the negative impact of partner phubbing on relational aggression in romantic relationships. On the other hand, partner phubbing reduces relational aggression by increasing support discrepancy. In the present study, the value of the support discrepancy was positive; that is, the actual support (*M* = 52.49) was less than the ideal support (*M* = 56.88). This result indicates that a wider gap between ideal and actual support (i.e., a greater degree of support discrepancy) may suppress the negative impact of partner phubbing on relational aggression, which is consistent with expectancy violation theory (Burgoon, 1993). When people show phubbing behavior in social situations, their behavior deviates from expectations (Miller-Ott and Kelly, 2015), for example, by constantly checking their phones without eye contact or by failing to respond immediately to each other. When the interaction in a romantic relationship cannot meet (or lags far behind) expectations, the relationship becomes unsatisfactory, which may lead to depression (Wang et al., 2020) and further reduce the interaction between couples. That’s why that support discrepancy was negatively associated with relational aggression. And thus, reducing the level of difference between actual and ideal support would decrease the negative impact of partner phubbing on relational aggression in romantic relationships.

Previous studies have emphasized the function of significant others in social support (e.g., Cantor and Little, 1985) and the essential role of the social support of partners in mental health (Hobfoll and Vaux, 1993). The present study provides a new perspective for understanding the role played by the social support of partners in romantic relationships, highlighting the importance of actual support and of the difference between ideal and actual support (i.e., support discrepancy). Although there is still a lack of intervention research on relational aggression in romantic relationships (Wang and Guoliang, 2019), this study provides possible targets for intervention to reduce relational aggression from the perspective of social support (i.e., actual support and support discrepancy) in young adults.

It should be noted that the correlation between partner phubbing and ideal support was not significant among both male and female participants. Partner phubbing behaviors will not directly affect partner’s expectations of support for the other party but may increase the support discrepancy by reducing actual support, so to some extent, ideal support still plays a role. Previous study showed that emotional support plays a strong role in determining satisfaction with a romantic relationship (Cramer, 2004), perhaps partner phubbing may be related to some important factors such as reduced emotional support. This study excluded ideal support from the mediation model, future research should further explore the mechanism of ideal support in the occurrence and development of partner phubbing and relational aggression.

### 4.3 Gender difference in the mediation model

The current study revealed that actual support and support discrepancy partially mediated the relationship between partner phubbing and relational aggression in females, but not in males; H4 is supported. Previous research has shown that females get more support from their surroundings, including support from significant others (Zhang et al., 2015), and that they use social support as a coping strategy more than males do (Cameron et al., 1996). Females are also more likely to engage in relational aggression than males (Goldstein et al., 2008); that is, when females have been phubbed by their partners, they are more likely to reduce social support for their partners, which leads to higher levels of relational aggression. For males, in contrast, a partner’s phubbing behavior is less likely to affect relational aggression indirectly via a reduction in actual support and an increase support discrepancy. The theory of social information processing emphasizes the role of cognition in aggressive behaviors, believing that a person’s response to setbacks or obvious provocations does not rely too much on the actual social cues presented, but depends on how they process and interpret this information (Terzian et al., 2015). In romantic relationships, females are more sensitive to male’s phubbing behavior and are more likely to interpret it as a relational aggression. They are more likely to punish their partners’ phubbing behavior, for example, criticizing them (Ren et al., 2022).

Compared to men, women are significantly more likely to receive affective support, such as confiding in each other, reassuring each other, and talking when upset (Lowenthal and Haven, 1968). Several studies have found that social support is associated with a series of psychological outcomes, including relational aggression. For instance, the availability and adequacy of the perceived support provided by a partner in the form of face-to-face communication can lead to lower levels of relational aggression (Nelson et al., 2008). Future research should explore in greater detail the mechanisms of the gender differences in this mediation model. Interventions and prevention measures for improving social support for young female adults in romantic relationships may help to reduce relational aggression.

### 4.4 Limitations and future research

There are several limitations of this study. First, the participants were recruited from an online survey in Mainland China, and so the generalizability of the findings to other regions and countries is limited. Second, the data were self-reported, which might introduce reporting bias in relation to the social desirability of certain responses, especially for young adults in a romantic relationship asked about their partner’s misbehavior in terms of phubbing and relational aggression. What’s more, this study focused on unmarried young adults and did not investigate other groups of age, which needs further research, Third, the survey was cross-sectional. Longitudinal studies are warranted to evaluate the causal relationships among the variables. Fourth, perpetrators and victims of relational aggression represent two different roles within romantic relationships, but we did not use them as two outcome variables to construct mediation model, respectively. In the future, we can further explore the differences and mechanisms between them. Finally, it is both possible and necessary to investigate other potential mediators and moderators in the association between partner phubbing and relational aggression. Previous studies suggest that partner phubbing not only evokes senses of resentment and jealousy from the phubbee (Krasnova et al., 2016), but also impels them to re-appraise their satisfaction of the romantic relationship (Wang et al., 2024), possibly leading to retaliation responses (Thomas et al., 2022).

## 5 Conclusion

The present study contributes to our understanding of the relationship between partner phubbing and relational aggression in young Chinese adults. Partner phubbing and social support may play essential roles in relational aggression. Given the high prevalence of phubbing behavior and the importance of romantic relationships in early adulthood, effective prevention and interventions are necessary. Meanwhile, measures to increase social support may help prevent relational aggression.

## Data Availability

The raw data supporting the conclusions of this article will be made available by the authors, without undue reservation.

## Footnotes

^1^ www.wjx.cn
